# Going Viral: Researching Safely on Social Media

**DOI:** 10.2196/29737

**Published:** 2021-12-13

**Authors:** Kari Dee Vallury, Barbara Baird, Emma Miller, Paul Ward

**Affiliations:** 1 College of Medicine and Public Health Flinders University Bedford Park Australia

**Keywords:** cyber bullying, online bullying, research activities, occupational safety, research ethics, students, bullying, social media

## Abstract

Safety issues for researchers conducting and disseminating research on social media have been inadequately addressed in institutional policies and practice globally, despite posing significant challenges to research staff and student well-being. In the context of the COVID-19 pandemic and given the myriad of advantages that web-based platforms offer researchers over traditional recruitment, data collection, and research dissemination methods, developing a comprehensive understanding of and guidance on the safe and effective conduct of research in web-based spaces has never been more pertinent. In this paper, we share our experience of using social media to recruit participants for a study on abortion stigma in Australia, which brought into focus the personal, professional, and institutional risks associated with conducting web-based research that goes viral. The lead researcher (KV), a postgraduate student, experienced a barrage of harassment on and beyond social media. The supportive yet uncoordinated institutional response highlighted gaps in practice, guidance, and policy relating to social media research ethics, researcher safety and well-being, planning for and managing web-based and offline risk, and coordinated organizational responses to adverse events. We call for and provide suggestions to inform the development of training, guidelines, and policies that address practical and ethical aspects of using social media for research, mental and physical health and safety risks and management, and the development of coordinated and evidence-based institutional- and individual-level responses to cyberbullying and harassment. Furthermore, we argue the case for the urgent development of this comprehensive guidance around researcher safety on the web, which would help to ensure that universities have the capacity to maximize the potential of social media for research while better supporting the well-being of their staff and students.

## Introduction

Social media is rapidly becoming a mainstream tool for the conduct and dissemination of research, health interventions, and evaluations [1]. Researchers and research students are increasingly expected to conduct and communicate their research on the web [2], including using a range of social media platforms to conduct and promote their work. Such spaces present new opportunities and risks for research. Rapid and potentially targeted recruitment and (perceived) anonymity provide access to historically hard-to-reach populations. At the same time, the boundaries between researchers’ professional and personal identities have become increasingly blurred as images, information, and work are shared and searchable across platforms. As such, communication with and harassment of researchers on the web can move rapidly from public to private spaces, with a suite of personal and professional consequences that are in line with those of web-based bullying and trolling more broadly.

In this context of new risks and opportunities, research ethics processes, the literature, and guidelines are beginning to address the specific concerns associated with research participant safety and well-being in web-based and social media research. However, robust and constructive cross-institutional and interdisciplinary conversations and guidance addressing the management of and support for researcher safety and well-being continue to be largely missing. In this paper, we argue that there is an urgent need for robust guidance on the use of social media for research, paying particular attention to the need for institutional and ethical frameworks and researcher training that address web-based safety and mental well-being. By outlining our extraordinary and challenging experience of *going viral*, along with the limited published experiences of other researchers, this paper calls for institutional and industry-wide practices that aim to keep researchers and their work safe in increasingly unavoidable web-based workspaces.

### New Norms, New Risks

Under consistent pressure to meet research performance expectations in the context of time constraints, in the COVID-19 pandemic environment of limited travel and face-to-face engagement opportunities, and given the benefits of engaging with technological innovations to improve research processes, researchers increasingly occupy web-based networks and social media platforms for the communication and conduct of research. In this context, social media–enabled recruitment has never been more relevant. The reach, speed, affordability, flexibility, and potential for multidirectional communication and *sharing features* afforded by social media make it a favorable alternative to traditional research processes and their limitations [3-6]. In particular, social media has been found to be an effective tool in health research and promotion. Social media has been used to successfully recruit hard-to-reach populations and may be particularly “well-suited to research and practice on ‘taboo’ public health topics” [4], such as sexual health. This is partly because of the potential for anonymity on social media, along with the high number of young people present on these platforms [4,6-10]. Engaging research participants via social media can help to minimize research fatigue, facilitate engagement and retention of research participants, and contribute a richer data set than traditional methods can achieve on their own [5].

Along with these benefits, the limited (albeit growing) body of literature on using social media for research also describes challenges, including self-selection bias, engagement, and underrecruitment, along with a lack of control over the framing and sharing of content shared on the web [8,11,12]. Social media platforms have been described as *echo chambers*; users are constantly and progressively exposed to content aligned to their pre-existing belief systems, confirmation bias thus being a feature of social media use [13]. This allows for the specific targeting of messaging and advertisements beneficial to the conduct of science and health promotion; it also means politically charged or emotionally arousing content is most likely to spur engagement and *go viral* [13,14].

There are additional potential challenges associated with the use of social media in research. The absence of facial and social cues and gestures on the web that would otherwise be present in face-to-face interactions and the real or perceived anonymity that web-based interactions can afford increase the potential for interpersonal conflicts and escalation of arguments [15-17]. “Language truncation, the use of images and hashtags, results in inappropriate, inaccurate or mis-judged commentary in 140 characters” [18], which can affect the narrative that surrounds research shared on the web and limit the ability of researchers to control it [8]. Misinformation, misinterpretation, and misappropriation of research or research activities on the web could be described as somewhat of an inevitability, as is highlighted in the discussion of our own experience. Users’ perceived anonymity and strength in numbers also means that communication and harassment among users can escalate rapidly, shifting from public to private and professional to personal web-based spaces [17,19,20]. Harassment on the web is not new; however, cultural and technological changes are likely to increase the risks of experiencing harassment and the speed at which *cyber mobs* rally, posing evolving challenges to researcher privacy, safety, and well-being.

Despite the myriad of challenges it poses, social media will be increasingly used by researchers who will become fluent in navigating and imagining its potential. Concurrently, these researchers will inevitably face evolving and fluent forms of harassment. As such, there is an onus on higher education and research industries and institutions to assume greater responsibility for the well-being of staff and students on the web, supporting and equipping them with the tools needed to safely navigate and effectively use these platforms and appropriately responding when harassment occurs.

### Going Viral: Triumphs and Troubles

As part of the primary author’s (KV) PhD research on abortion stigma in Australia, Facebook was used to recruit members of the Australian public to a web-based survey.

A number of professional, academic, and ethical challenges were faced by our research team during this process, which we share here in the hope that they will inform conversation and debate around the role of universities in better understanding, mitigating, and addressing researcher and student safety on the web.

Over 2 years, the authors developed a quantitative survey measuring abortion attitudes, knowledge, and perceived abortion stigma, which is the first of its kind to be developed and implemented in Australia. The survey tool was informed by extensive literature searching and qualitative and quantitative testing. It included, among others, a combination of items that endorsed and rejected stigmatizing abortion-related statements. The study received approval from the Flinders University ethics committee, including approval to omit all researcher names from the study documents.

Participants were recruited to the study using Facebook advertisements, which were targeted broadly at anyone living in Australia aged ≥16 years. Our ability to alter and retarget advertisements over time to ensure that the self-selected sample was as representative of the population as possible, the team’s familiarity with using paid Facebook advertising and the relative speed at which recruitment could occur made recruitment via Facebook an appealing and logical choice. It may be relevant to consider that the survey was released during the height of the first round of the COVID-19 pandemic restrictions in Australia in April 2020 when other methods of recruitment were likely to be more challenging than usual.

In just 2 weeks of Facebook advertising, 3500 participants completed the survey. At this time, the advertisements were retargeted to facilitate the recruitment of participants aged >40 years and male participants, underrepresented among the respondents. During the process of releasing these more targeted advertisements, the survey attracted the attention of a prominent antiabortion (prolife) lobby group who shared it with their membership via email and on their Facebook page. Within 48 hours, >5000 survey responses and close to 100 emails were received by the lead researcher (KV). At this time, the paid Facebook advertisements were halted, although the survey link remained live.

Comments undermining and debating the survey method and style, along with common antichoice sentiments around the “irresponsibility of women seeking abortion” and “abortion as murder” were noted as relevant social media posts. Emails to the research team and the university ethics committee contained *concerned queries* and *recommendations* for improvements, along with explicit hostility and requests to have the study ceased. McPherson et al [21] found that users who are the first to share a study (on social media) are likely to affect the composition of the resulting sample, reflecting the power and influence of individuals to amplify and influence messaging and information accuracy on the web. Our experience supports their finding, as the vast majority of the 5000 responses received in the days following the lobby group’s sharing of the study reflected their otherwise minority (in Australia) strong antiabortion views.

Coordinated attempts by this lobby group to undermine rights or evidence-based laws, policies, or programs, such as those pertaining to abortion, contraception, and lesbian, gay, bisexual, transgender, queer, intersex, and asexual (LGBTQIA+) rights, including marriage equality, are common in Australia [22,23]. Along with the use of more formal lobbying channels, direct communication (often abusive) with staff involved in projects or organizations that the group does not agree with have been reported [24,25]. The potential for such a response to our research was likely amplified by the growing (at least in prominence) public mistrust in science more broadly. This was facilitated by social media and exemplified by apparent global shifts towards conservatism over recent years [18], along with the abortion decriminalization process that fueled antiabortion activism in South Australia at the time our research was taking place.

Although it has been difficult to formally track *shares* and *reposts* of the study, 3 days after the survey was shared by the antiabortion group, a prominent feminist politician, feminist author and public figure, and a number of women’s health and women’s rights organizations became aware of the study. To counter the perceived attempts by antiabortionists to sway the findings, these individuals and groups began sharing the survey in their networks. The survey was subsequently shared at least several thousand times across Twitter and Facebook and emailed to multiple women’s health, women’s rights (feminist), health provider, and lobby group mailing lists across a 4-day period. Much of the narrative around these *shares* sought to encourage people to complete the survey to balance the nature of responses received. However, in a number of social media posts, the survey purpose was misconstrued as being a tool for promoting an antichoice agenda, causing anger from proponents of abortion rights. Items asking participants to select their level of agreement or disagreement with statements reflecting common abortion-related stereotypes and antiabortion sentiments were construed as evidence that the survey was inherently antichoice, which further fueled this narrative. As such, hostility from both proponents and opponents of abortion rights was directed at the research team and the university.

*Going viral* resulted in 67,000 responses in 6 days, with a total of 70,051 responses received over the 3-week recruitment period. Ultimately, the final sample broadly represented the Australian public regarding support for or opposition to abortion accessibility and legality, with approximately 89% (9/10) supporting legal abortion always or mostly [26,27]. The survey link was made inactive after a week of *going viral*, 3 weeks after it was first published, as the responses received represented a mix of views and were deemed more than sufficient to facilitate a detailed and meaningful analysis. Within days of ending the recruitment, the antichoice lobby group *claimed victory* in their email newsletter, suggesting it was their campaign against the study that resulted in it being closed.

A month later, a freedom of information request was submitted to the university to seek documents related to the study, including documents that indicated the reasons for the survey being closed and the survey responses themselves. As the lead researcher (KV) was a student, their name and most of the information requested were redacted. Details regarding other members of the research team and the content of several personal emails between the lead researcher and her supervisors were provided; some of them were later published on the web by the antiabortion lobby group.

Despite such a successful recruitment process, our unpreparedness for the speed with which the survey would be shared on the web led to a number of challenges for the research team. For example, we were initially unprepared to manage (practically and emotionally) the hundreds of hostile emails, which appeared to be a coordinated attempt to shut down the project and were received in a span of a few days. Although the researchers’ names were not in the public sphere, staying anonymous was a short-term solution, with the need to publish the work and findings, along with the freedom of information request, making disclosure inevitable.

A number of safety concerns arose, including concerns and uncertainty around the following: best practices for keeping safe on the web and preventing disclosure of personal details and location (of residence, in particular), the safety precautions that ought to be considered or implemented offline, and a lack of institutional capacity to provide such knowledge and support, the research team awareness of other strategies (with associated risks) that lobby or activist groups were likely to engage in, ways to balance the potential professional benefits of media interest with researcher and student well-being, and an understanding of risks and managing them to protect the university and individual reputations.

Phone and web-based meetings with the research team (because of the COVID-19 pandemic restrictions), on-campus phone-based mental health support, and the university media team and ethics committee were all available to support and respond to the lead researcher’s (KV) questions throughout the process of *going viral*. Although the responses received from individuals within the university were unanimously supportive, they were also ad hoc and sometimes conflicting. A coordinated response across departments, from media to ethics and student support, would have been beneficial in bolstering a sense of safety and clarity around how to respond and manage risks in relation to social media commentary, media requests and email communications, and threats.

Although it was deemed unlikely that web-based harassment would translate into offline risks of violence, a history of hostile activism and violence against abortion providers and supporters by antiabortion individuals and groups, both locally and abroad [28-30], contributed to heightened anxiety and fear throughout the experience. Recently, Glenza [31] described the antiabortion movement in the United States as *radicalized* and posing an *increased threat* [31]. Similarly, the decriminalization process and surrounding antiabortion campaign that occurred in South Australia, where the research team was located during the time of the research, heightened perceived risks. Overexposure to unpleasant social media commentaries and emails and comments on social media calling on people to *inundate* the research team with *concerned emails* resulted in the lead researcher (KV) experiencing both short- and long-term mental health consequences.

### Researcher Harassment on the Web: An Anomaly?

There is a dearth of literature documenting research *going viral* and its impact on research outcomes and researcher well-being. Kosinski et al [6] described a project that, owing to web-based snowball sampling, successfully recruited 6 million participants over 4 years, with safety concerns not reported. Cuevas [19], a social scientist in the United States, described his experience of a large-scale, coordinated harassment campaign. It began in response to a comment Cuevas [19] had made on a social media post regarding the 2016 US presidential campaign, which rapidly moved into private and personal messages, threats, racist slurs, and false reviews, resulting in coordinated attempts to undermine his employment and family well-being. Cuevas [19] filed police charges, and the harassment was treated as a hate crime; however, he continued to experience harassment and threats to his job security. Cuevas [19] published about his experiences in the hope of giving a “voice to others who have been similarly harassed,” stating in a media interview that he later received “emails from more than 60 professors from all over the world telling stories of their own” [32]. An Australian academic and antiracism activist, Dr Stephen Hagan, has also reported receiving hate mail and death threats in response to fake news reports about his work in advocating for the renaming of consumable products with racist connotations. Similar to that experienced by Cuevas [19], this hate campaign was fueled by right-wing political campaigns with racist dynamics [33]. Although neither of these harassment campaigns was initially in direct response to research activities, they were in response to web-based communication regarding their areas of expertise; in the Cuevas [19] case, the harassment rapidly became about his role as an academic and threatened it. As Viney [34] described, “academics have privileged knowledge that should be put to use in the community in a form of ‘ethical academia’.” As such, activism and academia are often fundamentally intertwined. As social media becomes a vital stage for the performance and communication of science and research, the relevant social media posts made by academics may be necessarily considered to be part of their work.

Other researchers have reported harassing experiences in response to Facebook advertising, including in response to advertisements for LGBTQIA+ research participants [20,35]. Mitchell and Jones [20] reported cyberbullying in the form of Facebook comments, private messages, and voicemails to their research team, demonstrating the way harassment moves effortlessly from public to private spaces. Researchers working with marginalized communities or on marginalized social issues are most likely to face web-based harassment (usually not originating from the marginalized communities in which they are working). Research has also found that “harassment often arises in spaces known for their freedom, lack of censure, and experimental nature” [36]. This suggests that there is a particular risk for academics who are inherently working in *experimental* spaces (ie, conducting research) and who may be conducting research with or are members of marginalized communities themselves.

*Trolling*, defined as web-based behavior deliberately intended to antagonize or offend someone [37,38], is often intended as a silencing strategy, as was much of the response to our abortion stigma work. However, trolling is not the only method used to silence victims of web-based harassment and abuse. The advice offered to victims to help them cope with trolling is often to *not engage* with or *further provoke* abusers. However, such advice further silences the voices of victims and their stories and is situated within a victim-blaming narrative, whereby conducting work on the web is in itself deemed a *provocation* and harassment a normal response [36].

Among the Australian public, negative web-based experiences are common. In 2019, 14% of adults in Australia were estimated to have been the target of hate speech [39], and 67% had negative experiences on the web [40]. Studies with university students internationally report varied rates of cyberbullying, in part likely because of definitional and measurement variations; however, it is common for such studies in the United States, Canada, and Australia to find that between 20% and 40% of participants have experienced cyberbullying [41].

Cyberbullying and harassment result in social, mental, physical, financial, and academic consequences for victims, and these impacts are more commonly experienced by minority or marginalized individuals and communities [16,17,39,42]. *Secondary traumatic stress* may be of particular concern for researchers witnessing harassment of their target populations or for those who have experienced personal trauma themselves [35]. Studies that have addressed cyberbullying in universities (investigating contexts of web-based learning and web-based bullying of staff by students or colleagues) have found that it can lead to significant psychological harm (in terms of mental health, productivity, and engagement), occupational impacts (including risks to job security, satisfaction, and employment opportunities), and physical consequences (including the risk of violence) [17,19,41].

### Ethical Considerations: Something Is Missing

The literature describing the ethical challenges associated with social media use in research are rooted in traditional ethical frameworks, with a focus on participant safety and protection. Ethical dilemmas regarding the appropriateness of the use of social media users’ web-based data as *research data* and the automatic sharing of social media users’ web-based behavior (including engagement with research-related content) with data companies are being increasingly addressed as they pertain to issues of consent, anonymity, and privacy [8,12]. Privacy and confidentiality risks to consenting research *participants* and nonparticipatory bystanders and the implications for participant *aftercare* (ie, the need for researchers to remain available to participants post data collection) have also been described [8,12,43]. Issues of inclusion and accessibility have also been raised, with the *digital divide* continuing to signal and exclude already-disadvantaged communities [5].

Ethics committees routinely request, as they must, detailed information about potential risks to participant safety and strategies to manage these risks. However, what is often neglected in ethics processes, the published literature on social media–based research, and institutional policies is researcher safety and well-being on the web. We acknowledge that this gap exists within a broader gap regarding researcher safety issues, described most frequently as relating to fieldwork and sensitive research, which are not new but remain inadequately addressed [5,44].

### A Call for Guidance and Integrated Management of Researcher Safety on the Web

Health and social scientists and research students can face considerable risks and consequences associated with conducting research on politically contested or otherwise sensitive topics, which are characteristic of many areas of health research [45,46]. However, such risks, particularly their relevance in web-based settings, have been insufficiently acknowledged in the literature, policy, or practice. Researcher safety and work health and safety in research are most often defined in terms of risks of physical violence in field and laboratory work [47-49]. Cyberbullying policies and the literature focus largely on peer-to-peer or peer-to-staff (or vice versa) interactions.

There appears to be a dearth of comprehensive and integrated frameworks, training, and guidance for preparing research staff and students to implement and manage their work and safety on the web, both at the institutional and research levels [50]. There is limited evidence-based or regulatory guidance on the use of social media for research broadly [3,6,8,12]. This contributes to ambiguity around relevant ethical considerations and best practices, including how to interpret and apply existing ethics principles [51]. Guidelines published by The British Psychological Society note that exposure to distressing content, unsolicited attention or messages, or *derogatory attacks* may cause emotional distress and threaten researcher and institutional reputations as a result of web-based research [20]. However, descriptions of risks and implications of ethical considerations regarding public–private distinctions, confidentiality, and anonymity (among others) for researchers are not provided, nor is guidance on mitigating or managing risk and adverse events. The under- or overestimation of risks resulting from a lack of ethical and practical guidance for web-based research and inconsistent approval outcomes from ethics boards affects researchers’ ability to conduct ethical web-based research and may discourage social media use in research, resulting in lost opportunities [5,6].

Research from North American universities has found that over half of their faculty members are unsure whether there are resources available to support them if they experience web-based bullying; however, they believe universities should be responsible for preventing and stopping web-based bullying [17]. Although Cuevas [19] reported a *uniformly supportive* response from the faculty to the harassment campaign against him, he also noted that he would have preferred a more assertive organizational response that would call out his attackers and deter future harassment campaigns rooted in the use of *collective power* against a public minority figure. Our own experience mirrors that of Cuevas [19] as responses to our experience were *uniformly* and personally supportive; however, there was no sense that a broader institutional response or positioning against the harassment was considered. This leads us to consider whether a desire to appear *objective* (and, likely, to appease diverse funders) mutes what should be confident, evidence-based, inclusive responses by academic and scientific institutions toward homophobic, antichoice, or other hate-fueled harassment of their staff and students.

Research institutions have a duty of care toward staff and students and, as such, an obligation to develop and implement strategies to protect researchers in the diversity of their modern workspaces. Although universities in Australia are legally mandated to hold policies addressing cyberbullying of staff, similar policies are not legally required for students [41]. A study found that although approximately 70% of Australian universities have policies relating to *bullying via computers*, less than half indicate *support for victims of bullying*, and only 20% provide *advice for students about bullying* [41]. An analysis of 465 policies at Canadian universities conducted in 2015 found that only one-third referenced cyber behaviors, and few addressed the prevention of web-based harassment [52]. Furthermore, such policies tend to focus on cyberbullying among peers or colleagues and often fail to address web-based safety management more broadly.

Failing to remain current with and address web-based safety concerns is not unique to universities. The *Guide for Preventing and Responding to Workplace Bullying* by Safework Australia [53] acknowledges the health and safety risks of bullying but fails to mention web-based harassment or bullying at all. However, as thought leaders and public institutions, it is questionable whether these gaps in universities—organizations that are designed to lead in knowledge generation and translation—are acceptable any longer.

### Recommendations

In 2019, Russomanno et al [35] published what they described as “the first formal, safety and monitoring guidelines for researchers using social media” for recruitment, particularly of marginalized population group members. These guidelines recommend protection for both participants and researchers, with a focus on minimizing, managing, and addressing negative comments and cyberbullying. Recommendations include assigning research team members to regularly administrate and monitor recruitment posts; posting advertisements for at most a 1-week period (at a time) to minimize researcher burnout; using inclusion *and* exclusion terms to minimize negative responses; restricting who can respond to or comment on public pages; frequent reviewing of Facebook policies around privacy, profanity, and reporting before recruitment to reduce the burden on research staff and decrease users’ experiences of negative comments and bullying; screenshotting and reporting all negative interactions to internal review boards; organizing regular staff debriefs and team meetings to minimize compassion fatigue (secondary traumatic stress); and making a relevant referral to mental health services or resources for staff as needed [35]. Evidence also suggests that the use of both inclusion and exclusion terms when targeting Facebook advertisements could help to minimize the likelihood of cyberbullying toward both the study population and, presumably, web-based researchers [20,35]. It is the specifics of managing safety, such as those that we believe should be shared and understood widely across research institutions and ethics boards.

On the basis of our experiences, relevant guidance addressing researcher safety on the web could also speak to the following:

1. The need for the routine provision of evidence-based training in ethical issues in web-based research for both researchers and ethics committees; this could support increased confidence of institutional review boards and individual researchers in using social media research strategies effectively, along with encouraging the teaching of techniques to minimize the risk of exposure to potentially harmful content and responses
2. Information on and strategies addressing the blurring of private and professional boundaries on the web and changing notions of privacy, including the implications for researcher safety and security, and guidance on the responsibilities of institutions in cases where harassment occurs and may move through public and private spaces
3. Emphasis on the legal, practical, and ethical implications of working across various social media platforms
4. The need to understand, support, and strengthen the digital fluency and mental health risks and capacity of researchers to prevent, manage, and respond to potential harassment and bullying, including clear protocols for individual and institutional support and response when harassment does occur
5. Strategies for engaging with media, both in the more traditional sense of media training and in regard to responding and communicating on the web, ensuring such strategies are not centered around avoidance of social media or on a victim-blaming mentality
6. Understanding language use, inclusion and exclusion terms, and other platform-specific features that can help researchers to minimize risks associated with social media–based recruitment

Universities may also benefit from institution-wide efforts toward understanding and planning for the ways in which various departments and roles across the organization need to contribute to and work together toward coordinated and effective responses to adverse events.

There appears to be a consensus in the literature that guidance pertaining to web-based research ethics should be based on traditional ethical and well-being frameworks, partially to aid ethics bodies in their transition to assessing risks in these *new* web-based workspaces, particularly as overarching ethical concerns remain the same across the various locations of research [5,8,50,54]. However, the evolving risks, expectations around privacy, personal and professional boundaries, and ethical norms will necessarily generate new understandings and definitions of safety and require new applications and imaginations of existing ethical frameworks [50].

Instead of fearing the unknowns and risks of web-based research, the development of comprehensive guidance around web-based safety will help to ensure that universities and research groups have the capacity to maximize the potential of social media for research while better supporting the well-being of research staff and students. As such, we propose that the higher education sector, research institutions, and ethics bodies need to engage more fully with the emerging risks social media presents. When the potential benefits for the quality of research outcomes and for staff and student well-being are weighed against the risks of not better engaging with these issues, the urgency and importance of this work become clear.

## Abbreviations

LGBTQIA+: lesbian, gay, bisexual, transgender, queer, intersex, and asexual
